# Annual Report of the State Cancer Laboratory

**Published:** 1907-02

**Authors:** 


### Annual Report of the State Cancer Laboratory.

THE annual report of the State Cancer Laboratory, the work of which is done in the Gratwick Laboratory building on High street, Buffalo, has been submitted to Dr. Eugene Porter, State Commissioner of Health. While there is nothing startling in the nature of discovery the report is interesting showing as it does that the increase in cancer is persistent and progressive, as Dr. Roswell Park pointed out in 1898. The figures showing comparative death rates for cancer and tuberculosis in two different groups of three years are startling in themselves. They show that tuberculosis has decreased 4.9%, and that cancer has increased 25.4%. The report states that the evidence is now sufficient for the Department of Health of the State of New York, as the result of researches conducted in this laboratory, to recommend to all health officers of the State, the desirability of proper sterilization and disposal of all dressings of cancer cases, the fumigation and sterilization of rooms occupied by cancer patients.

Although there is little evidence that individuals associated with cancer cases are in any immediate danger of contracting the disease, it is extremely probable that the contagion of cancer can be transferred from cancer patients to their surroundings, where in course of time it assumes a form in which it is again capable of infecting susceptible individuals.

This last statement is one which, though not novel in any sense, places the seal of official laboratory approval upon the similar statements made by Dr. Irving P. Lyon, of Buffalo, in his report of six or seven years ago, following his very painstaking investigations concerning the so-called cancer houses.

The present report deals rather fully with the work done by Ehrlich, of Frankfort, and the Royal Cancer Research Fund in London, and requests that the $3,000 which was cut out of the appropriation last year be given to the laboratory in this year's budget. Commenting on the question of finance the report says:

> In this connection I desire to call attention to the fact that the principals in the laboratory have, from the beginning, served it with great devotion on salaries which are not commensurate with their standing in the scientific world. The direction which cancer research has necessarily taken in the last two years necessitates the maintenance of a large staff of assistants, and with our present appropriation we are forced to employ a staff of thirteen individuals.

The relative expenditure for stock, material and equipment has so greatly increased by the direction which our work ha necessarily taken, that the laboratory has from the first paid the lowest possible salaries to its employes. The great increase in the cost of living will make it necessary to remedy some of the greatest discrepancies in this connection during the coming year, unless the laboratory is to lose the services of some of its most important members.

The members of the staff are: H. R. Gaylord, director; G. H. A. Clowes, chemist; G. N. Calkins, biologist; F. W. Baeslack, assistant in biology and histology; C. A. Maclay, secretary; D. R. Averill, assistant in chemistry; F. A. Payne, janitor, and six assistants classed as laborers.

The expenditures for the past year were $17,748.13. The laboratory should have more money for research purposes and if one were inclined to criticise the report at all it could only be on the score of excessive and retiring modesty of its author in relation to money matters. Too long has unselfish and self-sacrificing science been the plaything of commerce, and the toy of the politician. The State of New York is not poor and now that it has been definitely and officially shown by figures, which cannot lie, that cancer is on the increase, added impetus is given to the work of research. The paltry $21,000 asked for is too small, far too small and insignificant an amount to meet the requirements of the staff of the State Cancer Laboratory as at present constituted, and particularly is this so when one seriously considers the amount of work being done in the institution. Not only is original research work being carried on night and day, but some one of the staff is closely and personally watching the progress of work in European laboratories much of the time. The State should appropriate whatever amount the members of the staff deem necessary for their requirements. To say the least it is humiliating for men of standing in the scientific world to be compelled to plead for increase of salary, to even beg for an increase of pay to meet the increased cost of living.

